# Consumption of Cherry out of Season Changes White Adipose Tissue Gene Expression and Morphology to a Phenotype Prone to Fat Accumulation

**DOI:** 10.3390/nu10081102

**Published:** 2018-08-16

**Authors:** Albert Gibert-Ramos, Anna Crescenti, M. Josepa Salvadó

**Affiliations:** 1 Nutrigenomics Research Group, Department of Biochemistry and Biotechnology, Universitat Rovira i Virgili (URV), Tarragona 43007, Spain; mariajosepa.salvado@urv.cat; 2 Eurecat, Centre Tecnològic de Catalunya, Unitat de Nutrició i Salut, Reus 43204, Spain

**Keywords:** photoperiod, seasonality, obesity, cafeteria diet, adipose tissue, fruit consumption, xenohormesis, cherry

## Abstract

The aim of this study was to determine whether the consumption of cherry out of its normal harvest photoperiod affects adipose tissue, increasing the risk of obesity. Fischer 344 rats were held over a long day (LD) or a short day (SD), fed a standard diet (STD), and treated with a cherry lyophilizate (CH) or vehicle (VH) (*n* = 6). Biometric measurements, serum parameters, gene expression in white (RWAT) and brown (BAT) adipose tissues, and RWAT histology were analysed. A second experiment with similar conditions was performed (*n* = 10) but with a cafeteria diet (CAF). In the STD experiment, Bmal1 and Cry1 were downregulated in the CHSD group compared to the VHSD group. Pparα expression was downregulated while Ucp1 levels were higher in the BAT of the CHSD group compared to the VHSD group. In the CAF-fed rats, glucose and insulin serum levels increased, and the expression levels of lipogenesis and lipolysis genes in RWAT were downregulated, while the adipocyte area increased and the number of adipocytes diminished in the CHSD group compared to the VHSD group. In conclusion, we show that the consumption of cherry out of season influences the metabolism of adipose tissue and promotes fat accumulation when accompanied by an obesogenic diet.

## 1. Introduction

The obesity epidemic has become a worldwide problem over the last few decades caused by factors such as changes in lifestyle, including an increase in highly caloric food intake and a reduction in physical activity. However, scientists now agree that the reason for the rise in the number of obese people is much more complex, and that other factors, such as the duration of sleep at night and control over ambient temperature, may influence these numbers [1,2].

The adipose tissue of seasonal animals has been demonstrated to be directly influenced by the photoperiod or season [3,4]. Humans are also affected by seasonal changes, which affect body fat mass, activity, or the concentration of hormones all year long [5,6,7]. These changes are principally generated by the molecular clock [8], which is a set of autoregulatory loops generated in the suprachiasmatic nucleus (SCN) that regulates many physiological mechanisms in organisms, synchronizing the metabolism with the light–dark cycles of the environment, which are also known as the photoperiod. This mechanism is especially important in seasonal animals, which adapt in advance to the coming season to increase their chances of survival or reproduction [9,10]. Apart from the central clock in the SCN, peripheral tissues also present circadian rhythms, which follow the signalling provided by the central clock. In fact, molecular clock gene expression has been reported to follow a coordinated expression in white adipose tissue (WAT) during the day [11], and these expression levels have been reported to change depending on the rat photoperiod [12,13]. Interestingly, the time of food intake and high-fat diets, among other factors, have been shown to disrupt the molecular and peripheral clocks from the light–dark cycle, increasing the risk of obesity and other diseases [14,15,16,17,18]. In this sense, it has been shown that polyphenols, secondary metabolites found in some vegetables and fruits, are capable of entraining the peripheral clocks in lean and obese rats [19].

Related to these secondary metabolites is the xenohormesis theory, which posits that some phytochemicals produced by plants or other autotrophs can have a direct effect on the enzymes and receptors of heterotrophs caused by common evolutionary conserved signalling pathways [20]. These molecules produced by plants have been demonstrated to change depending on the season, the environment, and different stresses [21,22,23]. Thus, this signalling could be useful for animals, in that they would be able to anticipate large changes in the environment or changing seasons and develop suitable survival adaptations [20]. In this regard, a healthy approach to counteract the obesity epidemic has been to increase the amount of fruits and vegetables in the diet [24]. However, in the globalized world, it is increasingly common to purchase seasonal fruits all year long. For example, cherry is a fruit harvested between spring and summer, but now, due to international trade, it is possible to purchase cherry produced in the other hemisphere during autumn or winter, or it can be grown locally in the natural growing season and then be stored and eaten in another season. All these factors, together with the xenohormesis theory and the disturbances on the molecular clocks described above, give rise to a new question about whether fruit from a determinate season, which contains a particular molecular signature of the environment and season, consumed at another time of the year could affect the molecular clocks and/or increase the risk of obesity.

The hypothesis of our study was that the consumption of fruit from a photoperiod different from the one an animal is acclimated to could send incorrect signals to the animal, which will either develop characteristics of another photoperiod or season or increase the risk of obesity due to desynchronization of the molecular clock. Both WAT and brown adipose tissue (BAT) are central to obesity. WAT relates to obesity mainly due to its specialization in the accumulation of a surplus of energy in the form of fat [25]. BAT is involved in energy expenditure because it expresses uncoupling protein 1 (Ucp1), which is capable of uncoupling the proton gradient from the synthesis of ATP in mitochondria, generating heat in the process [26]. For these reasons, we focused this work on the adipose tissue, and we developed this idea first with animals fed a standard diet (STD) to study how the normal adaptations of Fischer 344 rats to the photoperiod might change when cherry is consumed out of season. Furthermore, we performed a second experiment with cafeteria (CAF)-fed rats, which increases their caloric consumption with a highly palatable diet, in order to study how these changes might be modified in an obesogenic environment.

## 2. Materials and Methods

### 2.1. Treatments

Sweet cherry (*Prunus avium* L.) was of the Royal Dawn variety and purchased at Mercabarna (Barcelona, Spain). Cherry fruit was frozen in liquid nitrogen, ground with a blender, and freeze-dried with a lyophilizer. Afterwards, the powder was stored and protected from light at room temperature until use. The phenolic composition of sweet cherries was 171.42 mg/100 g fresh weight of fruit (FW) anthocyanins; 15.07 mg/100 g FW flavanols; and 87.81 mg/100 g FW phenolic acids. A more detailed description of the composition is included in the Supplemental Data. The nutritional (Table S1) and phenolic (Table S2) compositions were obtained from the U.S. Department of Agriculture [27] and Phenol Explorer [28], respectively.

We supplemented rats with an oral dose of 100 mg/kg body weight of cherry lyophilizate (CH), which in a human of 70 kg would be equivalent to 42.6 g of raw cherries or a small portion of fruit, which is below the standard daily recommendation of the World Health Organization (WHO) [29]. According to the WHO [30], the daily sugar intake for humans should not surpass 10% of their total energy intake. For this reason, we selected a dose that, in conjunction with the average daily intake of sugar included in the standard diet, would not surpass the 10% limit indicated by the WHO.

The vehicle (VH) treatment consisted of a 1:1 glucose:fructose solution in water with a concentration that ranged from 20 mg/mL to 33 mg/mL during the experiment depending on the volume to be administered and the rat weight so as not to administer an excessive volume to the animal. This solution was used to match the sugar consumption with that of the cherry (CH) treatment.

### 2.2. Animal Experimental Procedure

**Figure 1:**
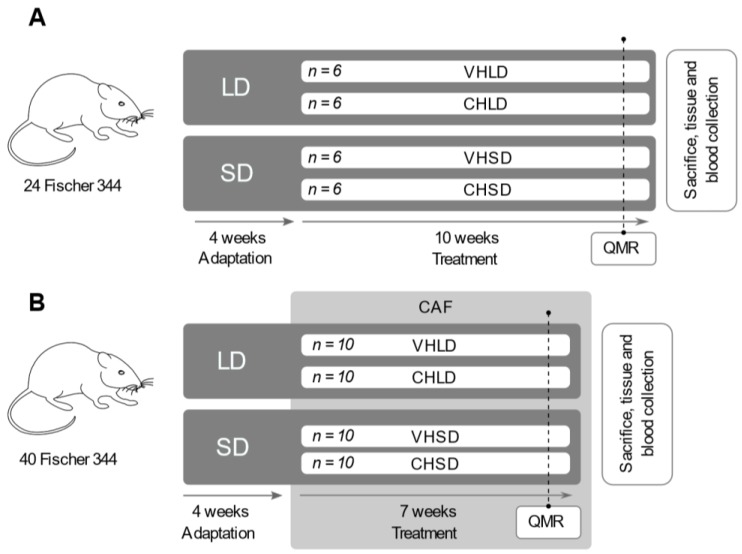
Experimental design of the two studies. For the first experiment (**A**), 24 Fischer 344 rats fed with a standard chow diet were randomly distributed into four groups (*n* = 6) and acclimated to a long (LD) or a short (SD) photoperiod (18 h light: 6 h dark; 6 h light: 18 h dark, respectively) over 4 weeks. After the adaptation period, animals were treated with 100 mg/kg body weight of cherry lyophilizate (CH) or 20 mg/kg of the vehicle solution (VH) for 10 weeks. One week prior to sacrifice, fat mass and lean mass were analysed by quantitative magnetic resonance (QMR). For the second experiment (**B**), 40 Fischer 344 rats were distributed into four groups (*n* = 10) and adapted in the same conditions as the first experiment. After the adaptation, animals were fed a cafeteria diet (CAF) and treated daily with the same dose and vehicle of the first experiment for 7 weeks, and 1 week prior to sacrifice, fat mass and lean mass were measured by QMR.

For the first experiment, we used 24 2-month-old male Fischer 344/IcoCrl rats (Charles River Laboratories, Barcelona, Spain) fed with a standard chow diet (STD) (Panlab, Barcelona, Spain) with a caloric distribution (3.2 kcal/g) of 19.3% protein, 8.4% fat, and 72.4% carbohydrates. The animals were housed two per cage at 22 °C and 55% humidity and with free access to food and water. The animals were randomly distributed into four groups (*n* = 6) depending on the treatment received and the photoperiod to which they were exposed. Animals were acclimated to two photoperiods with different light:dark cycles: long-day (LD, 18 h light:6 h dark) and short-day (SD, 6 h light:18 h dark) over 4 weeks. After the adaptation period, animals in each photoperiod were treated daily with 100 mg/kg body weight of CH or with 20 mg/kg body weight of the VH for 10 weeks. Accordingly, the four animal groups of the study were CHLD (cherry, long day), CHSD (cherry, short day), VHLD (vehicle, long day), and VHSD (vehicle, short day) (Figure 1A).

For the second experiment, 40 Fischer 344/IcoCrl rats were fed a CAF diet ad libitum, consisting of bacon (8–12 g), biscuits with pâté (12–15 g) and cheese (10–12 g), muffins (8–10 g), carrots (6–9 g), and sweetened milk (22% sucrose *w*/*v*; 50 mL) in addition to the same standard chow diet of the first experiment [31]. The caloric distribution of the CAF diet (5.28 kcal/g) was 10% protein, 31.9% fat, and 58.1% carbohydrates. The food was freshly provided daily. The nutritional composition of the STD and CAF diets is described in the supplementary material (Table S3). This diet model induces hyperphagia of highly caloric ingredients, which increases fat and sugar ingestion to develop the main features of metabolic syndrome and obesity [32]. Before starting the experiment, the animals were randomly distributed into four groups (*n* = 10) depending on the treatment received and the photoperiod to which they were exposed. As in the first STD experiment, animals were acclimated to the two photoperiods, LD and SD, for 4 weeks. After the adaptation period, animals in each photoperiod were fed a CAF diet and treated daily with 100 mg/kg body weight of CH or with 20 mg/kg body weight of the VH for seven weeks. Accordingly, as in the first experiment, the four animal groups of the study were CHLD, CHSD, VHLD, and VHSD (Figure 1B).

The body weight and food intake of animals were recorded every week for both experiments. One week prior to sacrifice, the fat mass and lean mass were analysed by quantitative magnetic resonance using an EchoMRI-700™ (Echo Medical Systems, LLC., Houston, TX, USA) without anaesthesia. Animals were then sacrificed in the fed state by decapitation, and blood was collected from the neck, stored at room temperature for 45 min, and then centrifuged at 1200× *g* for 10 min to collect the serum. Different white adipose tissue deposits—epididymal (EWAT), retroperitoneal (RWAT), inguinal (IWAT), and mesenteric (MWAT)—and interscapular BAT were rapidly removed after death, weighed, frozen in liquid nitrogen, and then stored at −80 °C until further analysis. We chose to sacrifice the animals in the fed state because this simulates more precisely the conditions found in humans, who spend the majority of the day in the postprandial state [33,34]. This allows for the study of the energetic metabolism of the adipose tissue, specifically lipid uptake and lipogenesis, as it shows higher activity levels responding to the increased glucose and lipid content in plasma [35,36].

Adiposity was determined by an adiposity index computed for each rat as the sum of the EWAT, IWAT, MWAT, and RWAT deposit weights and expressed as a percentage of total body weight.

The Animal Ethics Committee of the University Rovira i Virgili (Tarragona, Spain) approved all of the procedures (reference number 4249), and the guidelines for the use and care of laboratory animals of the university were followed.

### 2.3. Plasma Analysis

Enzymatic colorimetric kits were used for the determination of plasma glucose (Ref. 992320) and triglycerides (Ref. 998282), (QCA, Barcelona, Spain). Insulin (Ref. EZRMI-13K) and leptin (Ref. EZML-82K) levels were quantified with a rat-specific enzyme immunoassay kit (Millipore, Madrid, Spain).

### 2.4. RNA Extraction and Quantification by Real-Time qRT-PCR

Total RNA from RWAT and BAT tissues was extracted using Trizol^®^ reagent (Ambion, Life Technologies, Uppsala, Sweden) following the manufacturer’s instructions. The RNA yield was quantified with a Nanodrop ND-1000 spectrophotometer (NanoDrop Technologies, Wilmington, DE, USA), and the integrity of the RNA was confirmed using agarose gel electrophoresis.

In brief, 0.5 µg of total RNA was reverse-transcribed using a High-Capacity cDNA Reverse Transcription Kit (Applied Biosystems, Madrid, Spain) in a Multigene Thermal Cycler (Labnet, Madrid, Spain), and for Q-PCR, the CFX96 real-time system C1000 Touch Thermal Cycler (Bio-Rad, Barcelona, Spain) with the iTaq™ Universal SYBR^®^ Green Supermix (Bio-Rad, Barcelona, Spain) was used. All Q-PCRs were performed with the following cycling conditions after an initial Taq activation at 95 °C for 30 s: 39 cycles of 95 °C for 5 s and 60 °C for 30 s. A melt curve was produced after the previous steps by increasing the temperature from 65 °C to 95 °C by 0.5 °C every 5 s. Gene expression levels in RWAT tissue were determined for the acetyl-CoA carboxylase alpha (*Acacα*), fatty acid synthase (*Fasn*), glycerol-3-phosphate acyltransferase (*Gpat*), monoglyceride lipase (*Mgll*), adipose triglyceride lipase (A*tgl*), hormone-sensitive lipase (*Hsl*), CCAAT/enhancer-binding protein alpha (*C*/*ebpα*), peroxisome proliferator-activated receptor gamma (*Pparγ*), brain and muscle ARNT-like1 (*Bmal1*), cryptochrome circadian clock 1 (*Cry1*), and period circadian clock 2 (Per2) genes. In BAT tissue, we measured gene expression levels for the cluster of differentiation 36 (*Cd36*), fatty acid transport protein 1 (*Fatp1*), lipoprotein lipase (*Lpl*), carnitine palmitoyltransferase 1B (*CPT1b*), hydroxyacyl-CoA dehydrogenase (*Had*), and peroxisome proliferator-activated receptor alpha (*Pparα*) genes. Furthermore, we measured the gene expression levels of PR domain containing 16 (*Prdm16*) and uncoupling protein 1 (*Ucp1*) in both tissues. The primers for the different genes are described in the Supplemental Data (Table S4) and were obtained from Biomers.net (Ulm, Germany). The relative expression of each mRNA was calculated as a percentage of the vehicle group using the 2^−∆∆Ct^ method [37] with *Ppia*, *Actb*, and *Hprt* as reference genes. Each qRT-PCR was performed at least in duplicate.

### 2.5. Histology

For histological analyses, frozen RWAT samples were thawed and fixed in 4% formaldehyde. The tissue underwent successive dehydration series and was afterwards embedded in paraffin (Citadel 2000, HistoStar, Thermo Scientific, Madrid, Spain). Paraffin blocks were cut into 2-μm-thick sections using a microtome (Microm HM 355S, Thermo Scientific). The sections were subjected to automated haematoxylin–eosin staining (Varistain Gemini, Shandom, Thermo Scientific) [38].

The sections were observed, and images were acquired at ×10 magnification using the AxioVision Zeiss Imaging software (Carl Zeiss Iberia, S.L., Madrid, Spain). The area and number of adipocytes were measured using the open source software Adiposoft (CIMA, University of Navarra, Navarra, Spain). Four fields per sample and 6 samples from each group were measured. The area was calculated from the average value of the area in all measured fields for each group. The total adipocyte number was calculated using the formula $(\frac{\pi }{6})\;x\;\left(3\sigma^{2}\;x\;\bar{d}+{\bar{d}}^{3}\right)$, where $\bar{d}$ is the mean diameter and σ is the standard deviation of the diameter, to obtain the average adipocyte volume [39]. Afterwards, we converted this value to the average adipocyte weight using the adipocyte density (0.92 g/mL) and, to obtain the total adipocyte number, the weight of the IWAT deposit was divided by the average adipocyte weight as proposed by *Lemmonier* [40]. Frequencies of adipocytes were obtained by distributing cells into two groups according to their area (<5000 µm^2^ or >5000 µm^2^) and calculated as a percentage of the total number of counted cells.

### 2.6. Statistical Analysis

The software IBM SPSS (SPSS Inc., Chicago, IL, USA) was used for statistical analysis. Data are expressed as the mean ± standard error of the mean (SEM), and significant differences were analysed by a one-way ANOVA test followed by Duncan’s new multiple range test for post hoc comparison between all groups. A *p* value ≤ 0.05 was considered statistically significant.

## 3. Results

### 3.1. Biometric Parameters

In the first experiment with rats fed the STD, there were no significant changes in weight or cumulative caloric intake between groups. Similarly, no changes were detected among the adipose deposits for fat and lean mass or in the plasma parameters between the VH and CH groups (Table 1).

The second experiment with rats fed the CAF diet also showed no differences in weight between groups. However, the CHLD group showed a significantly lower caloric intake than that of the VHLD group (Table 2). Regarding the weight of the different adipose tissue deposits, no differences were observed between groups. Concerning plasmatic parameters, the CHSD group had significantly higher glucose and insulin serum levels compared to those in the VHSD group (Table 2).

### 3.2. RWAT Gene Expression

**Figure 2:**
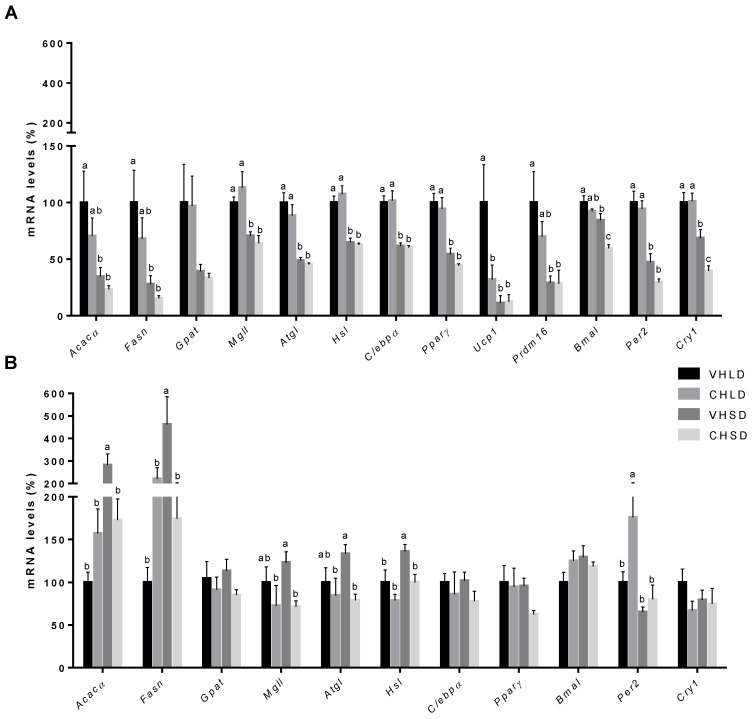
mRNA expression levels in RWAT of Fischer 344 rats supplemented with a cherry (CH) lyophilizate or vehicle (VH), held in long-day (LD) and short-day (SD) photoperiods, and fed either with a standard (STD) (**A**) or a cafeteria (CAF) (**B**) diet in two independent experiments. Expression of genes related to lipogenesis, lipolysis, adipogenesis, thermogenesis, and the molecular clock. Data are presented as the ratios of gene expression relative to *β-actin*, *Ppia*, and *Hprt* genes and expressed as a percentage of the LD group set at 100%. Data are presented as the mean ± SEM, and the four groups were compared with a one-way ANOVA test (*p* < 0.05) followed by a Duncan’s new multiple range test (MRT) post hoc test. ^abc^ Mean values with unlike letters differ significantly among groups.

In the STD experiment, Ucp1 expression was significantly downregulated in the CHLD group compared to that in the VHLD group. The clock genes, Bmal1 and Cry1, were significantly downregulated in the CHSD group compared to those in the VHSD group. No differences between groups were found among the other analysed genes (Figure 2A).

In CAF-fed rats, the expression levels of the genes Acacα and Fasn, which code for lipogenic enzymes, and of the genes *Mgll*, *Atgl*, and *Hsl*, which code for lipolytic enzymes, were significantly decreased in the CHSD group compared to those in the VHSD group. Instead, the transcriptional modulator Per2 was significantly upregulated in the CHLD group compared to that in the VHLD group. The expression levels of *Ucp1* and of *Prdm16*, the transcriptional coregulators that control the development of brown adipocytes, were undetectable on each of the studied groups (Figure 2B).

### 3.3. RWAT Histology

**Figure 3:**
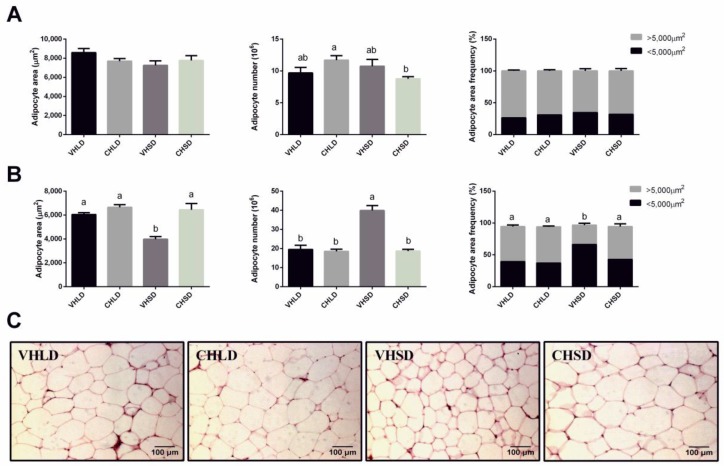
Adipocyte area, adipocyte total number, and adipocyte area frequencies of RWAT of Fischer 344 rats supplemented with a cherry (CH) lyophilizate or vehicle (VH), held in long-day (LD) and short-day (SD) photoperiods, and fed either with an STD (**A**) or a CAF (**B**) diet in two independent experiments. Representative pictures of all the CAF groups are shown (**C**). For frequencies, adipocytes were distributed into two groups depending on their areas (<5000 or >5000 µm^2^). Data are presented as the mean ± SEM, and the four groups were compared with a one-way ANOVA test (*p* < 0.05) followed by a Duncan’s new multiple range test (MRT) post hoc test. ^ab^ Mean values with unlike letters differ significantly among groups.

No differences were found in the morphology of the RWAT between the VH and CH groups of rats fed the STD diet (Figure 3A). However, rats fed the CAF diet and subjected to an SD photoperiod showed a significantly higher adipocyte area and a smaller number of adipocytes in the CHSD group than that in the VHSD group. In addition, the CHSD group showed a significantly increased frequency of larger adipocytes compared to that in the VHSD group (Figure 3B).

### 3.4. BAT Gene Expression

**Figure 4:**
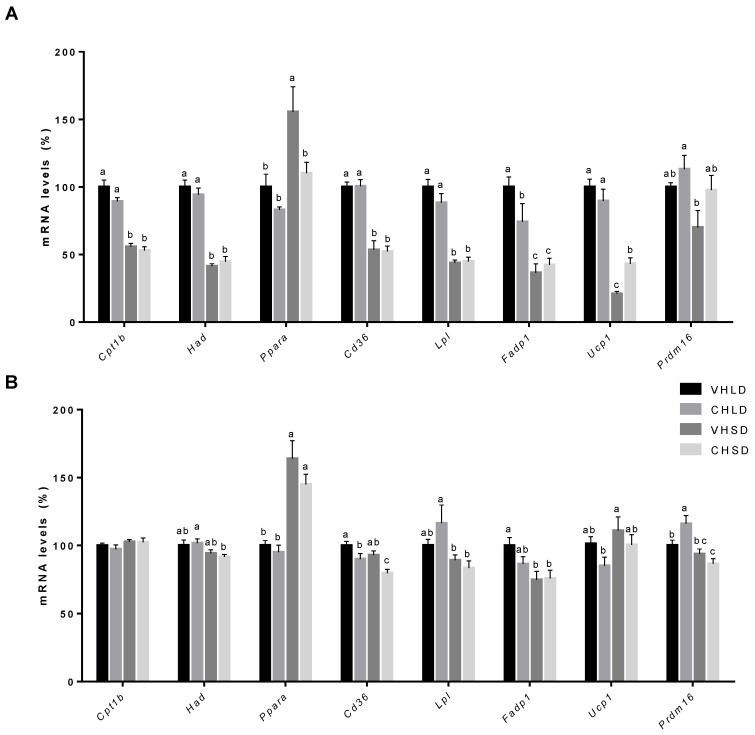
mRNA expression levels in BAT of Fischer 344 rats supplemented with a cherry (CH) lyophilizate or vehicle (VH), held in long-day (LD) and short-day (SD) photoperiods, and fed either with an STD (**A**) or a CAF (**B**) diet in two independent experiments. Expression of genes related to lipogenesis, lipolysis, adipogenesis, and thermogenesis. Data are presented as the ratio of gene expression relative to the *β-actin*, *Ppia*, and H*prt* genes and expressed as a percentage of the LD group set at 100%. Data are presented as the mean ± SEM, and the four groups are compared with a one-way ANOVA test (*p* < 0.05) followed by a Duncan’s new multiple range test (MRT) post hoc test. ^abc^ Mean values with unlike letters differ significantly among groups.

In the STD experiment, we observed a significant downregulation of the nuclear receptor involved in BAT fatty acid uptake and β-oxidation, *Pparα*, and an upregulation of *Ucp1* in the CHSD group compared to that in the VHSD group (Figure 4A).

In the CAF experiment, differences were found in the fatty acid translocase Cd36, which was significantly downregulated in both CH groups compared to that in the VH groups. *Prdm16* expression showed a statistically significant upregulation in the CHLD group compared to that in the VHLD group (Figure 4B).

## 4. Discussion

An increasing number of new factors that could contribute to the obesity epidemic are being found. For example, it is known that alterations in the photoperiod, such as those observed in night-shift workers, are well-correlated with an increased risk of obesity [15,41]. In this study, we wanted to focus on a brand-new aspect that could act as a risk factor in the development of obesity, namely the consumption of out-of-season fruit.

As observed in previous results from our group [4] and data from other authors, Fischer 344 rats adapt to changes to the photoperiod for reproductive purposes [42,43], and in an LD they tend to increase body weight by either increasing lean [42] or fat mass [44]. These characteristics are typical of seasonal animals, which detect the changes in light and dark during the seasons and adapt accordingly, generally increasing fat reserves during an LD and depleting them during an SD [3]. Furthermore, according to the xenohormesis theory, plants synthetize secondary metabolites depending on diverse exogenous factors or stresses, such as water availability, light, and temperature [21,22,23], so it is understandable that this molecular signature will be different depending on the environment. Moreover, it has been found that these phytochemicals, such as polyphenols, can entrain the molecular clock [19], and thus we hypothesized that they might desynchronise or somehow modify the normal response of animals depending on the photoperiod where they were consumed.

In STD-fed rats, we did not observe biometric changes apart from those between the photoperiod groups, which have been already described by *Gibert-Ramos* et al. [4]. In the RWAT, the CHLD group showed a decrease in the expression level of *Ucp1* compared to that in the VHLD group. *Ucp1* is a good marker of browning of WAT [45], so this downregulation could mean that the ingestion of cherry in an LD photoperiod decreases the thermogenesis in WAT. Even so, these results were not conclusive, since we detected no other changes that would suggest an inhibition of the browning or the thermogenesis in the WAT, such as an increase in adiposity or in adipocyte size, among others. Furthermore, and contrary to what we found, there is evidence in the literature that some polyphenols might promote the activation of BAT [46] and the browning of WAT [47,48,49]. In fact, vanillic acid, a metabolite of anthocyanins, which are found in cherry, has been reported to promote the browning of the WAT in mice, increasing the gene expression of *Ucp1* and *Prdm16* [50].

In STD-fed animals, CHSD rats had decreased levels of *Pparα* and increased levels of *Ucp1* in their BAT. As we have already discussed in another manuscript [4], we have signs that *Pparα* is not carrying out its function, since we obtained contradictory results compared with other data. *Pparα* is a nuclear receptor that activates the transcription of genes related to fatty acid uptake and β-oxidation [51]. Additionally, it has been found that increased levels of *Pparα* activate *Ucp1* transcription [52], and thus we would expect higher levels in the VHSD group and not in the CHSD group. For these reasons, *Pparα* seems to incorrectly represent the metabolic state of BAT, possibly because of post-transcriptional or post-translational regulation, or because the retinoic X receptor (RXR), a ligand necessary for *Pparα*’s correct functioning, is not present [53]. Furthermore, there is evidence that RXR can control BAT development and activation [54], that RXR is needed for adipogenesis, and that the disruption of the RXR–*Pparα* heterodimer reduces adipocyte formation [55]. These hypotheses are supported by studies that demonstrate that *Pparα* regulates brown adipose tissue thermogenesis, activating *Ucp1* and *Prdm16* gene expression [52], which, in our study, were downregulated in the short-day (SD) groups versus the long-day (LD) groups together with the studied β-oxidation and lipid uptake genes. On the other hand, increased *Ucp1* levels could correspond to what we observed in the LD groups. Cherry is an LD fruit, so its consumption during an SD could erroneously signal the animal, which would develop adaptations typical of an LD.

In the CAF experiment, the ingestion of cherry in the LD showed a reduction in the accumulated caloric intake. Cherry, as with all vegetables and fruits, contains polyphenols [56,57], which have been reported to possess beneficial effects against obesity, including a reduction in food intake in some cases [58]. In order to obtain further information about the possible mechanisms by which CH ingestion could affect the physiology and seasonal adaptations of adipose tissue, it would be appropriate in future studies to characterize the polyphenolic metabolites in serum after its supplementation. The lack of caloric intake reduction in the CHSD group might be related to the consumption of cherry out of season, and thus the expected effects of cherry were not observed. Additionally, these changes were only observed in the CAF experiment and not the STD, probably because of their increased caloric intake, which makes changes more easily detected, and because of the pro-obesogenic environment of a highly palatable diet, which changes the whole system of food intake regulation in rats [59].

The CHSD group consumed cherry out of season, and according to our hypothesis, we would expect metabolic changes disassociated from the short photoperiod. These changes were observed first in the plasmatic parameters of CAF-fed rats, where insulin and glucose levels were significantly higher than those in the VHSD group. This increase in insulin circulation levels in a postprandial state is a response to increased glucose concentration, which is generally determined by the diet. However, in our study, no differences in caloric intake between the SD groups nor in carbohydrate consumption (data not shown) were found, so other mechanisms, including nutrient absorption in the intestine or metabolization, should be considered. Moreover, insulin levels in obese rats are higher than those in lean rats [60], and postprandial hyperglycaemia has been reported to be a predictor of diabetes [61], which suggests an increased predisposition of CHSD rats to obesity or a decreased sensitivity to insulin. Interestingly, CHSD rats showed an increase in adipocyte area compared to that in the VHSD group, while the total adipocyte number was decreased. Adipocyte hypertrophy has also been linked to type II diabetes and insulin resistance [62,63] and thus appears to indicate a higher predisposition of the LD groups and CHSD groups to these diseases when fed a CAF diet.

CH consumption also altered gene expression in the RWAT in the SD groups. While the VHSD group had higher expression levels of lipogenesis genes (*Acacα* and *Fasn)* than the LD groups, consumption of CH downregulated these genes, achieving similar levels to those of the LD groups. A similar effect was observed in *Mgll*, *Atgl*, and *Hsl*, which were downregulated in the CHSD group. *Auguet* et al. found that in morbidly obese patients, the fatty acid absorption and transport in the visceral WAT were downregulated [64]. Additionally, the authors also found a decrease in the gene expression levels of *Acacα* and *Fasn*, as in our study, and a decrease in their protein levels [64]. Other studies report that adipocyte size is directly implicated in the disruption of the adipocyte functionality of obese individuals [65]. Specifically, studies have reported that hypertrophic adipocytes in the WAT suffer a dysregulation of its ability to store and mobilize lipids, which decreases the gene expression of key genes of lipogenesis and lipolysis as we observed in our study [66,67].

Considering that a CAF modifies the normal effect of the photoperiod, as observed previously with the VH groups [4], the ingestion of CH, a fruit from an LD, appears to affect somehow the photoperiodic effect of the SD in the metabolism of the adipose tissue. Altogether, data in the CAF animals appear to suggest that rats consuming cherry in an SD develop a small degree of insulin resistance that might, or might not, be related to the similar changes observed in the LD groups. This change is further observed in the BAT, where the expression of *Cd36*, a key transporter of fatty acid and lipoprotein into the cell [68], was also downregulated in accordance with what we observed in the WAT. On the other hand, *Cd36* was also downregulated in the BAT of CHLD rats and thus might indicate a direct effect of cherry on the expression levels of this gene.

According to our hypothesis, the observed changes in our study could be promoted by a desynchronization of the peripheral clocks in the CHSD group. For this reason, we quantified the gene expression levels of three key genes implicated in the autoregulatory loop of the molecular clock—*Bmal1*, *Per2*, and *Cry1* [9]—which have been found to follow a coordinated expression in the adipose tissue during the day [11]. In our study, the STD-fed animals showed a clearly different pattern of expression between the LD and SD groups, which should be taken as the standard pattern of expression. However, this pattern was altered in the CHSD group of STD rats, which showed lower expression levels of *Bmal1* and *Cry1* compared to those of the other three groups, indicating a dysregulation of the clock machinery in the WAT produced by the CH consumption out of season. Nevertheless, these changes were not translated into any biometrical or physiological effect in the parameters we analysed.

On the other hand, CAF-fed rats showed no differences in the gene expression of the molecular clock between the VH groups. To our knowledge, this is the first time that a CAF diet was used to study the gene expression of the molecular clock in rats, and thus, as already discussed in [4], we should take into account that the CAF might be playing a desynchronizing role. This high-fat diet model was composed of highly palatable ingredients that increase food consumption in comparison to that of a chow diet [31], and it has been shown that feeding time and diet can disrupt the circadian system, with consequences for the development of obesity [17,18,69,70,71]. For these reasons, we believe that the CAF is desynchronizing the expression of *Bmal1*, *Per2*, and *Cry1*, so we are unable to appreciate the differences between the VHLD and VHSD groups. Concerning CH consumption, we observed a significant upregulation of *Per2* in the CHLD group compared to that in the VHLD and SD groups. However, none of the other two clock genes analysed showed significant changes, and thus we are unsure about how this result could have a significant effect on the other parameters analysed. To obtain more robust evidence of changes in the clock machinery in WAT, future studies should focus on obtaining data at different time points in order to compare the oscillations of expression levels of the clock genes during the day between groups [11].

## 5. Conclusions

In conclusion, we show that the consumption of cherry, a fruit that is harvested during spring or summer, and so, from a long-day season, affects the metabolism of the adipose tissue of Fischer 344 rats differently depending on the photoperiod in which it is consumed, affecting the physiology of the adipose tissue to one more prone to fat accumulation when consumed out of season. This study shows evidence about how fruit origin and seasonality might play a role in the risk of developing obesity in humans. Although more evidence is required, the results of this study could be useful for the development of obesity prevention diets.

## Figures and Tables

**Table 1:** Biometric and plasmatic measurements of rats supplemented with cherry or vehicle on long- and short-day photoperiods.

	VHLD	CHLD	VHSD	CHSD
Weight (g)	386.5 ± 12.66	388 ± 5.11	370.33 ± 10.99	376.5 ± 13.58
Accumulated caloric intake (kcal)	504.79 ± 11.67	493.3 ± 10.78	507.07 ± 9.44	498.44 ± 13.38
Fat (g)	55.64 ± 4.41 ^a^	58.38 ± 3.11 ^a^	45.06 ± 1.29 ^b^	50.41 ± 2.82 ^ab^
Lean (g)	309.74 ± 8.96	307.43 ± 4.59	295.76 ± 8.19	299.16 ± 11.89
Fat (%)	14.38 ± 0.75 ^ab^	15.17 ± 0.7 ^a^	12.52 ± 0.34 ^b^	13.43 ± 0.57 ^ab^
Lean (%)	80.71 ± 0.67	79.95 ± 0.76	80.94 ± 1.04	79.69 ± 0.62
EWAT (g)	12.02 ± 0.94 ^ab^	12.99 ± 0.7 ^a^	9.65 ± 0.67 ^b^	9.64 ± 0.71 ^b^
MWAT (g)	8.04 ± 0.85	7.95 ± 0.45	6.53 ± 0.82	6.42 ± 0.65
IWAT (g)	5.82 ± 0.73	6.27 ± 0.3	4.52 ± 0.53	5.66 ± 1.03
RWAT (g)	10 ± 0.67 ^ab^	10.33 ± 0.29 ^a^	8.52 ± 0.58 ^b^	8.71 ± 0.47 ^ab^
Adiposity Index (%)	9.22 ± 0.49 ^ab^	9.67 ± 0.34 ^a^	7.72 ± 0.47 ^b^	8.05 ± 0.55 ^b^
EWAT (%)	3.09 ± 0.17 ^a^	3.34 ± 0.16 ^a^	2.6 ± 0.14 ^b^	2.56 ± 0.16 ^b^
MWAT (%)	2.06 ± 0.17	2.05 ± 0.11	1.74 ± 0.16	1.69 ± 0.13
IWAT (%)	1.5 ± 0.15	1.62 ± 0.08	1.25 ± 0.14	1.49 ± 0.25
RWAT (%)	2.58 ± 0.11 ^ab^	2.66 ± 0.07 ^a^	2.41 ± 0.05 ^ab^	2.31 ± 0.09 ^b^
Glucose (mmol/L)	136.81 ± 3.48 ^ab^	133.61 ± 3.49 ^b^	139.24 ± 3.51 ^ab^	145.5 ± 2.72 ^a^
Triglycerides (mg/dL)	142.06 ± 7.74 ^bc^	120.03 ± 10.22 ^c^	197.9 ± 14.45 ^a^	181.22 ± 23.03 ^ab^
Insulin (ng/mL)	5.54 ± 0.73	6.15 ± 0.96	4.04 ± 0.66	4.03 ± 0.79
Leptin (ng/mL)	18.56 ± 0.31 ^ab^	22.69 ± 2.19 ^a^	16.59 ± 1.49 ^b^	16.21 ± 2.08 ^b^

Fischer 344 rats supplemented with cherry (CH) lyophilizate or vehicle (VH) on long-day (LD) and short-day (SD) photoperiods. Adiposity index was computed as the sum of epididymal white adipose tissue (EWAT), mesenteric white adipose tissue (MWAT), inguinal white adipose tissue (IWAT), and retroperitoneal white adipose tissue (RWAT) deposit weights and expressed as a percentage of total body weight. BAT, interscapular brown adipose tissue. Data are presented as the mean ± standard error of the mean (SEM) and the four groups were compared with one-way ANOVA (*p* < 0.05) followed by a Duncan’s new multiple range test (MRT) post hoc test. ^abc^ Mean values with unlike letters differ significantly among groups.

**Table 2:** Biometric and plasmatic measurements of rats supplemented with cherry or vehicle and a cafeteria diet in long-day and short-day photoperiods.

	VHLD	CHLD	VHSD	CHSD
Weight (g)	411 ± 7.77	422.89 ± 7.14	407.1 ± 12.03	404.3 ± 9.61
Accumulated caloric intake (kcal)	1364.34 ± 37.39 ^a^	1214.67 ± 58.02 ^b^	1298.52 ± 48.45 ^ab^	1198.2 ± 30.34 ^b^
Fat (g)	89.51 ± 3.8	86.56 ± 2.23	85.84 ± 3.27	88.29 ± 5.31
Lean (g)	291.95 ± 5.14	299.07 ± 5.23	294.5 ± 8.32	296.42 ± 3.82
Fat (%)	22.03 ± 0.63	21.7 ± 0.66	21.53 ± 0.79	21.82 ± 1.04
Lean (%)	72.07 ± 0.54	72.5 ± 0.62	73.68 ± 0.76	72.33 ± 1.07
EWAT (g)	16.23 ± 0.82	15.75 ± 0.53	14.75 ± 0.72	15.61 ± 1.08
MWAT (g)	9.42 ± 0.61	9.25 ± 0.66	8.68 ± 0.47	8.28 ± 0.69
IWAT (g)	11.9 ± 1.39	11 ± 1.02	12.47 ± 1.39	10.39 ± 1.25
RWAT (g)	13.53 ± 0.59	13.41 ± 0.26	12.87 ± 0.54	12.41 ± 0.59
Adiposity Index (%)	12.23 ± 0.54	11.51 ± 0.3	11.95 ± 0.42	11.5 ± 0.58
EWAT (%)	3.93 ± 0.13	3.76 ± 0.09	3.62 ± 0.14	3.84 ± 0.21
MWAT (%)	2.28 ± 0.11	2.18 ± 0.13	2.15 ± 0.14	2.03 ± 0.14
IWAT (%)	2.87 ± 0.31	2.59 ± 0.21	3.02 ± 0.27	2.57 ± 0.31
RWAT (%)	3.28 ± 0.1	3.18 ± 0.06	3.16 ± 0.08	3.14 ± 0.06
Glucose (mmol/L)	182.87 ± 4.65 ^ab^	180.5 ± 9.23 ^ab^	164.13 ± 4.91 ^b^	202.31 ± 15.46 ^a^
Triglycerides (mg/dL)	437.02 ± 30.47	421.07 ± 12.89	444.19 ± 42.34	449.75 ± 48.06
Insulin (ng/mL)	6.59 ± 0.47 ^ab^	7.42 ± 0.49 ^a^	5.82 ± 0.24 ^b^	7.31 ± 0.59 ^a^
Leptin (ng/mL)	19.89 ± 0.55	20.08 ± 1.08	21.41 ± 1.33	20.9 ± 0.96

Fischer 344 rats supplemented with cherry (CH) lyophilizate or vehicle (VH) and fed a cafeteria diet in long-day (LD) and short-day (SD) photoperiods. The adiposity index was computed as the sum of EWAT, MWAT, IWAT, and RWAT deposit weights and expressed as a percentage of total body weight. BAT, interscapular brown adipose tissue; EWAT, epididymal white adipose tissue; MWAT, mesenteric white adipose tissue; IWAT, inguinal white adipose tissue; RWAT, retroperitoneal white adipose tissue. Data are presented as the mean ± SEM, and the four groups were compared with a one-way ANOVA test (*p* < 0.05) followed by a Duncan’s new multiple range test (MRT) post hoc test. ^ab^ Mean values with unlike letters differ significantly among groups.

## Supplementary Materials

The following are available online at http://www.mdpi.com/2072-6643/10/8/1102/s1, Table S1: Nutritional composition of sweet cherry, Table S2: Phenolic composition of sweet cherry, Table S3: Nutritional composition of the standard and cafeteria diets, Table S4: Primers for the Q-PCR analysis.
