# Supplementary material for: Consumption of Cherry out of Season Changes White Adipose Tissue Gene Expression and Morphology to a Phenotype Prone to Fat Accumulation
Source: Nutrients. 2018 Aug 16;10(8):1102. doi: 10.3390/nu10081102 (PMC6115965; doi:10.3390/nu10081102)
Supplement: Supplementary file 1 [file nutrients-10-01102-s001.pdf]

Supplemental Table S1. Nutritional composition of sweet cherry

Proximates	Value per 100 g FW
Water (g)	82.25
Energy (kcal)	63
Protein (g)	1.06
Total lipid (fat) (g)	0.2
Carbohydrate, by difference (g)	16.01
Fiber, total dietary (g)	2.1
Sugars, total (g)	12.82
**Minerals**	
Calcium, Ca (mg)	13
Iron, Fe (mg)	0.36
Magnesium, Mg (mg)	11
Phosphorus, P (mg)	21
Potassium, K (mg)	222
Sodium, Na (mg)	0
Zinc, Zn (mg)	0.07
**Vitamins**	
Vitamin C, total ascorbic acid (mg)	7
Thiamin (mg)	0.027
Riboflavin (mg)	0.033
Niacin (mg)	0.154
Vitamin B-6 (mg)	0.049
Folate, DFE (µg)	4
Vitamin B-12 (µg)	0
Vitamin A, RAE (µg)	3
Vitamin A, IU (IU)	64
Vitamin E (alpha-tocopherol) (mg)	0.07
Vitamin D (D2 + D3) (µg)	0
Vitamin D (IU)	0
Vitamin K (phylloquinone) (µg)	2.1
**Lipids**	
Fatty acids, total saturated (g)	0.038
Fatty acids, total monounsaturated (g)	0.047
Fatty acids, total polyunsaturated (g)	0.052
Fatty acids, total trans (g)	0
Cholesterol (mg)	0

Nutritional composition of sweet cherry expressed for 100 g fresh weight (FW). Data obtained from the U.S. Department of Agriculture [1].

Supplemental Table S2. Phenolic composition of sweet cherry.

**Flavonoids**		**mg per 100 g FW**
Anthocyanins	Cyanidin 3-O-glucoside	18.73
	Cyanidin 3-O-rutinoside	143.27
	Pelargonidin 3-O-rutinoside	1.24
	Peonidin 3-O-glucoside	0.76
	Peonidin 3-O-rutinoside	7.42
Flavanols	(+)-Catechin	1.5
	(-)-Epicatechin	7.78
	(-)-Epicatechin 3-O-gallate	0.09
	(-)-Epigallocatechin	0.05
	Procyanidin dimer B1	0.23
	Procyanidin dimer B2	2.1
	Procyanidin dimer B3	0.08
	Procyanidin dimer B4	0.18
	Procyanidin dimer B5	0.2
	Procyanidin dimer B7	1.01
	Procyanidin trimer C1	1.85
**Phenolic acids**		
Hydroxycinnamic acids	3-Caffeoylquinic acid	44.71
	3-Feruloylquinic acid	0.43
	3-p-Coumaroylquinic acid	38.43
	4-Caffeoylquinic acid	0.77
	4-p-Coumaroylquinic acid	1.27
	5-Caffeoylquinic acid	2.2

Phenolic composition of sweet cherry expressed as mg/ 100 g fresh weight (FW).

Data obtained from Phenol explorer [2].

Supplemental Table S3. Nutritional composition of the standard and cafeteria diets.

	gr/100 gr of diet	
	**STD**	**CAF**
**Proteins**	16.1	5.8
**Lipids**	3.1	8.4
Of which saturated fatty acids	0.65	2.6
**Carbohydrates**	60.4	32.9
Of which total sugars	1.9	19.6
**Fibres**	3.9	1.8
**Moisture**	11.9	48.0

Nutritional composition of the standard (STD) and cafeteria (CAF). The sources of carbohydrates of the STD diet were 100% from cereals, and the protein sources were 66.7% from vegetal origin and a 33.3 % from animal origin. The ingredients of the cafeteria diet were bacon (8-12 gr), biscuits with pâté (12-15 gr) and cheese (10-12 gr), muffins (8-10 gr), carrots (6-9 gr) and sweetened milk (22% sucrose w/v; 50 mL) in addition to the standard chow diet.

Supplemental Table S4. Primers for the Q-PCR analysis.

	**Forward (5'...3')**	**Reverse (5'...3')**
Hprt	TCCCAGCGTCGTGATTAGTGA	CCTTCATGACATCTCGAGCAAG
Actb	GCAGGAGTACGATGAGTCCG	ACGCAGCTCAGTAACAGTCC
Ppia	CTTCGAGCTGTTTGCAGACAA	AAGTCACCACCCTGGCACATG
Acacα	GCGGCTCTGGAGGTATATGT	TCTGTTTAGCGTGGGGATGT
Atgl	GAAGACCCTGCCTGCTGATT	CACATAGCGCACCCCTTGAA
Fasn	TAAGCGGTCTGGAAAGCTGA	CACCAGTGTTTGTTCCTCGG
Gpat	GAATACAGCCTTGGCCGATG	GAGGCGTGCATGAATAGCAA
Hsl	AGTTCCCTCTTTACGGGTGG	GCTTGGGGTCAGAGGTTAGT
Prdm16	GTTCTGCGTGGATGCCAATC	TGGCGAGGTTTTGGTCATCA
Cebpα	TGTACTGTATGTCGCCAGCC	TGGTTTAGCATAGACGCGCA
Mgll	ATCATCCCCGAGTCAGGACA	TGACTCCCCTAGACCACGAG
Ucp1	GGTACCCACATCAGGCAACA	TCTGCTAGGCAGGCAGAAAC
Lpl	GGCCCAGCAACATTATCCAG	ACTCAAAGTTAGGCCCAGCT
Had	ATCGTGAACCGTCTCTTGGT	AGGACTGGGCTGAAATAAGG
Cpt1b	GCAAACTGGACCGAGAAGAG	CCTTGAAGAAGCGACCTTTG
Pparα	CGGCGTTGAAAACAAGGAGG	TTGGGTTCCATGATGTCGCA
Fatp1	CTACCACTCAGCAGGGAACA	GCGGCATATTTCACCGATGT
Cd36	CAGTGCAGAAACAGTGGTTGTCT	TGACATTTGCAGGTCCATCTATG
Pparγ	AGGGCGATCTTGACAGGAAA	CGAAACTGGCACCCTTGAAA
Bmal1	GTAGATCAGAGGGCGACGGCTA	CTTGTCTGTAAAACTTGCCTGTGAC
Cry1	TGGAAGGTATGCGTGTCCTC	TCCAGGAGAACCTCCTCACG
Per2	CGGACCTGGCTTCAGTTCAT	AGGATCCAAGAACGGCACAG

Hypoxanthine-guanine phosphoribosyltransferase (*Hprt*), Actin beta (*Actb*), Peptidylprolyl Isomerase A (*Ppia*), acetyl-CoA carboxylase alpha (*Acacα*), adipose triglyceride lipase (Atgl), fatty acid synthase (*Fasn*), glycerol-3-phosphate acyltransferase (*Gpat*), hormone-sensitive lipase (*Hsl*), PR domain containing 16 (*Prdm16*), CCAAT/enhancer-binding protein alpha (*C/ebpα*), monoglyceride lipase (*Mgll*), uncoupling protein 1 (*Ucp1*), lipoprotein lipase (*Lpl*), hydroxyacyl-CoA dehydrogenase (*Had*), carnitine palmitoyltransferase 1B (*CPT1b*), peroxisome proliferator-activated receptor alpha (*Pparα*), fatty acid transport protein 1 (*Fatp1*), cluster of differentiation 36 (*Cd36*), peroxisome proliferator-activated receptor gamma (*Pparγ*), brain and muscle ARNT-like1 (*Bmal1*), cryptochrome circadian clock 1 (*Cry1*) and period circadian clock 2 (*Per2*).

References

1. US Department of Agriculture, Agricultural Research Service, N. D. L. USDA Food Composition Databases Available online: https://ndb.nal.usda.gov/ndb/ (accessed on Jun 8, 2018).

2. Vos, F.; Crespy, V.; Chaffaut, L.; Mennen, L.; Knox, C.; Neveu, V. Phenol-Explorer : an online comprehensive database on polyphenol contents in foods. *Database Oxf.* **2010**, *2010*, 1–14, doi:10.1093/database/bap024.
